# Healthcare Access Through Digital Coordination: A Nationwide Analysis of Obstetrics and Gynecology E-Referral Patterns in Saudi Arabia

**DOI:** 10.3390/healthcare14070883

**Published:** 2026-03-30

**Authors:** Abdullah A. Alharbi, Meshary S. Binhotan, Mohammed A. Muaddi, Ahmad Y. Alqassim, Ali K. Alsultan, Mohammed S. Arafat, Abdulrahman Aldhabib, Yasser A. Alaska, Eid B. Alwahbi, Afnan Alomar, Rakan Saleh Al-Rasheed, Nawfal A. Aljerian

**Affiliations:** 1 Department of Family & Community Medicine, College of Medicine, Jazan University, Jazan 45142, Saudi Arabia; aaalharbi@jazanu.edu.sa (A.A.A.); aalqassim@jazanu.edu.sa (A.Y.A.); 2 Emergency Medical Services Department, College of Applied Medical Sciences, King Saud bin Abdulaziz University for Health Science, Riyadh 11481, Saudi Arabia; hotanm@ksau-hs.edu.sa (M.S.B.); dr.rocky20@gmail.com (R.S.A.-R.); njerian@moh.gov.sa (N.A.A.); 3 King Abdullah International Medical Research Centre, Riyadh 11481, Saudi Arabia; 4 Department of Emergency Medicine, King Abdulaziz Medical City, Riyadh 14611, Saudi Arabia; 5 Medical Referrals Centre, Ministry of Health, Riyadh 12382, Saudi Arabia; alkalsultan@moh.gov.sa (A.K.A.); arafatms@moh.gov.sa (M.S.A.); aaldhabib@moh.gov.sa (A.A.); ealwahbi@moh.gov.sa (E.B.A.); 6 Department of Emergency Medicine, King Saud University, Riyadh 11461, Saudi Arabia; yalaska@ksu.edu.sa; 7 Department of Obstetrics and Gynecology, Jazan General Hospital, Jazan Health Cluster, Jazan 82723, Saudi Arabia; fnooo-wbs@hotmail.com

**Keywords:** care coordination technology, coordinated care pathways, digital health platforms, healthcare coordination, healthcare delivery, women’s health

## Abstract

**Highlights:**

**What are the main findings?**

- Analysis of 39,526 OB/GYN e-referrals through the Saudi Medical Referrals Centre digital platform revealed a 91.81% overall acceptance rate nationwide.
- Subspecialty-specific referral patterns showed that obstetrics and fetal medicine had the highest proportion of urgent referrals (58.85%) and NICU bed utilization (55.62%).

**What are the implications of the main findings?**

- National e-referral data can characterize subspecialty-specific utilization patterns across large geographic regions, providing a foundation for understanding the needs of OB/GYN subspecialties.
- E-referral data provide valuable insights for digital health integration, strategic subspecialty planning, and healthcare access.

**Abstract:**

**Background/Objectives:** Obstetrics and gynecology healthcare represents a global health concern requiring coordinated, accessible services across diverse populations. The Saudi Medical Referrals Centre (MRC) functions as a comprehensive digital health surveillance and coordination platform managing nationwide obstetrics and gynecology (OB/GYN) services. This study characterizes national OB/GYN e-referral patterns coordinated through the MRC platform to describe subspecialty utilization and inform capacity planning, and examines temporal trends in referral direction over the study period. **Methods:** A retrospective descriptive analysis of the MRC’s digital coordination platform examined 39,526 OB/GYN referrals across Saudi Arabia’s healthcare system (2023–2024). Descriptive statistics, chi-square tests, and one-way ANOVA tests were used to analyze patient demographics, subspecialty distribution, referral types, bed requirements, acceptance rates, geographic patterns, and multivariable logistic regression examined temporal trends in referral direction. **Results:** The platform achieved 91.81% overall acceptance rates. Platform surveillance revealed referral request distribution by subspecialty: general OB/GYN (60.68%), obstetrics and fetal medicine (16.37%), and reproductive endocrinology and infertility (14.94%). Most referral requests were for outpatient care (71.35%), though obstetrics and fetal medicine demonstrated relatively high NICU utilization (55.62%). Urgent referral requests constituted 22.05% of cases. Internal referral odds increased 1.7% monthly over the study period (OR = 1.017; *p* < 0.001). **Conclusions:** This nationwide descriptive analysis of 39,526 OB/GYN e-referrals reveals distinct subspecialty-specific referral patterns, with high overall acceptance rates and predominantly internal referral coordination. These system-level findings provide a baseline for future studies within digital referral platforms.

## 1. Introduction

Improving maternal health has been a global health concern for decades [1,2]. While maternal mortality has decreased over the last two decades, the mortality rate remains high, with many pregnancies experiencing potentially life-threatening complications, necessitating further improvement [3]. Despite this, the majority of maternal deaths stem from preventable and treatable obstetric-related causes such as hemorrhage, sepsis, and obstructed labor [4,5]. However, obstetrics cases are distinct, and the management of their complications requires immediate coordinated access to healthcare services staffed by specialized personnel and equipped with the necessary resources (e.g., neonatal intensive care units (NICU)) [4,5]. Delays in coordinating access to obstetric complications care can intensify these conditions, potentially resulting in the death of both the mother and the fetus [6,7,8].

Furthermore, women’s health is significantly influenced by gynecological-related disorders, affecting their quality of life [9,10]. These diseases are prevalent and represent a major cause of disability-adjusted life years among women of reproductive age [11], with little progress made in reducing the burden over the years [12]. Gynecological conditions can include urinary incontinence, female infertility, endometriosis, and various gynecological tumors [13,14]. Some conditions may have limited specialized facilities available, while others require the expertise of highly specialized professionals and advanced care settings such as intensive care units (ICU) [15,16]. As the field of obstetrics and gynecology (OB/GYN) advances, several subspecialities have been introduced to address these complex disorders, including reproductive endocrinology and infertility, maternal–fetal medicine, female pelvic medicine and reconstructive surgery, and gynecologic oncology [17,18]. However, accessing this specialized care remains a challenge due to factors such as a shortage of specialists, geographical barriers, and financial constraints [19,20,21]. A well-established referral system can enhance access to this specialized care.

The Kingdom of Saudi Arabia has deployed a nationwide coordination e-referral system called the Medical Referrals Centre (MRC) [22]. This system integrates all governmental and the majority of private hospitals in one platform, to improve communication, data exchange, and decision-making for referral requests across all medical specialties. MRC services cover both rural and urban areas across Saudi Arabia, coordinating both internal referrals—where the referring and receiving health facilities are in the same region—and external referrals—where these facilities are located in different regions [23,24]. This system demonstrated significant improvements in enhancing coordinated access to patients in need of different healthcare services, including intensive care units, mental health services, and other specialized care [22,23,24,25,26]. OB/GYN referral coordination is one of the services offered by MRC; however, national-level data on OB/GYN subspecialty-specific referral volumes, acceptance rates, and geographic distribution through this platform have not been characterized, limiting evidence-based subspecialty capacity planning.

With the rapid expansion of specialized women’s health services across Saudi Arabia, a comprehensive analysis of national OB/GYN referral patterns through MRC provides a unique opportunity to examine subspecialty utilization on an unprecedented scale. This study aimed to characterize subspecialty-specific referral patterns, acceptance rates, and geographic distribution of OB/GYN e-referrals coordinated through the MRC platform from 2023 to 2024. The findings will elucidate distinct referral profiles by subspecialty, identify capacity demands, and reveal care coordination patterns to inform strategic development of women’s healthcare infrastructure and specialized human resources. By providing comprehensive surveillance data on national OB/GYN referral utilization, this study aims to support evidence-based capacity planning and optimize coordinated access to quality specialized OB/GYN care for women throughout Saudi Arabia.

A referral system aims to provide access to the required healthcare resources that are not available at a patient’s initial access point [27]. An effective referral system has been shown to grant immediate access, thereby improving patient outcomes [28]. E-referral systems have demonstrated potential to improve referral quality, reduce incomplete information, and shorten waiting times [28]. Systematic reviews across specialties further show that digital referral interventions, particularly organizational changes such as e-consultation and triage modification, can enhance provider communication and decrease referral delays [29,30]. Existing studies have largely focused on primary-to-secondary care transitions in cardiology, dermatology, and orthopedics, with limited attention to multi-subspecialty coordination [29,30]. Evidence on digital platforms coordinating the full breadth of OB/GYN subspecialties at a national scale remains lacking. While referring OB/GYN cases is vital to provide access, some studies revealed a lack of effective implementation of the referral method with significant delays within the referral process [31,32]. This gap is particularly relevant given that OB/GYN coordination must accommodate both urgent maternal–fetal transfers and elective subspecialty consultations with distinct clinical prerequisites. Employing an efficient and well-structured electronic referral (e-referral) system can enhance communication and data exchange, and therefore promote timely access to healthcare services [28].

To date, no studies have examined the performance of national e-referral systems in coordinating OB/GYN subspecialties, limiting policymakers’ ability to make informed decisions regarding capacity planning and resource allocation. To address this gap, the present study characterizes national OB/GYN e-referral patterns coordinated through the Saudi Medical Referrals Centre (MRC) platform, describing subspecialty utilization, acceptance rates, and geographic patterns and examining temporal trends in referral direction to provide baseline data for strategic planning of women’s healthcare services aligned with Saudi Vision 2030.

The conceptual framework for this study is based on the Donabedian Model comprising structure, process, and outcome (SPO) components [33]. In this framework, structure encompasses MRC’s digital infrastructure, including the electronic referral platform, integrated hospital networks, and centralized database systems connecting healthcare facilities nationwide. Process involves MRC’s coordination mechanisms that facilitate communication between referring and receiving hospitals, classify cases by urgency level (routine outpatient, urgent, routine inpatient, lifesaving, and services), and match patients with appropriate subspecialty services through electronic referral submission. Outcome reflects system-level performance measured through referral acceptance rates, with cases directed internally within regions or externally to other institutions based on service availability, bed capacity, and subspecialty expertise. This framework provides the analytical lens through which the study’s descriptive findings are organized and interpreted.

## 2. Methods

### 2.1. Study Design and Setting

This nationwide retrospective analysis of the MRC administrative database examined OB/GYN e-referrals processed through the digital health surveillance and coordination platform from January 2023 to December 2024. The MRC system coordinates all OB/GYN referrals using a Unified System of Medical Referrals (USMR), which hospitals can access digitally to request a referral.

**Figure 1:**
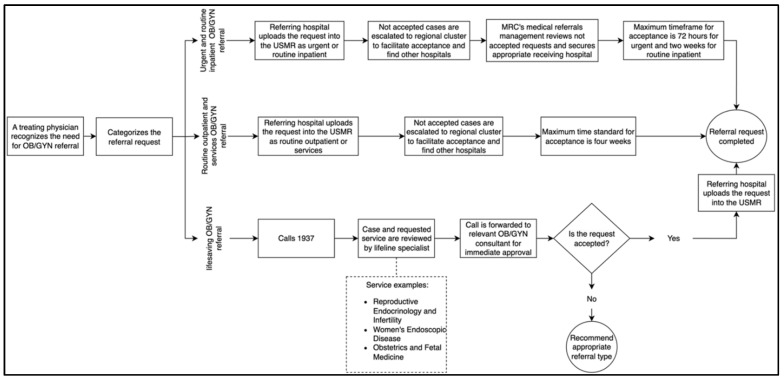
The Process of Initiating and Accepting Obstetrics and Gynecology E-referral Requests Through the Saudi Medical Referrals Centre E-referral System.

Any treating physician can request a referral through the e-referral system when a need arises. The USMR offers different types of referrals, developed based on scientific criteria, which are lifesaving, urgent, routine inpatient, routine outpatient, and services. The referral request is categorized into one of these types based on the treating physician’s assessment of the patient’s medical condition, following the MRC’s protocol for these categories (Figure 1). For urgent and routine inpatient referrals, requests are uploaded by the referring hospital into the e-referral system and sent to potential receiving hospitals. If there is no acceptance, the requests are escalated to the regional cluster to facilitate the acceptance process and find other potential hospitals. Unaccepted requests are then reviewed by the MRC’s medical referrals management to secure an appropriate hospital. For routine outpatient and services referrals, requests are uploaded into the e-referral system and forwarded to a potential receiving hospital. The regional cluster reviews the non-accepted cases to secure acceptance from other hospitals.

MRC also offers a 24 h hotline ‘1937’ service for lifesaving referrals, to expedite the referral acceptance for critically ill patients. The call is made by the treating physician and answered by a lifeline specialist for assessment, and then forwarded to a relevant OB/GYN consultant for a comprehensive review and immediate acceptance. Approved requests are provided with a receiving hospital name and uploaded into the USMR by the referring hospital, while those not approved are advised to be uploaded into the system with the recommended referral type. During the referral process, all patients continue to receive the necessary medical management until they are transferred to the receiving facility.

The study included all OB/GYN specialty referrals across Saudi Arabia’s healthcare network, which is administratively organized into five business units (BUs): Central (Riyadh and Al-Qassim), Eastern (Eastern region), Western (Makkah, Madinah, and Al-Baha), Northern (Al-Jouf, Northern Borders, Tabuk, and Hail), and Southern (Asir, Jazan, and Najran) [34].

### 2.2. Data Source and Collection

This study analyzed 39,526 OB/GYN e-referral records extracted from the MRC surveillance and coordination platform. Initially, 40,748 records were extracted. Each patient referral is assigned a unique code upon system upload, which may be sent to multiple hospitals simultaneously to expedite acceptance. Once a hospital accepts, all other referrals with that code are automatically stopped, and the accepted referral is retained while duplicates are deleted. Conversely, if all hospitals reject the referrals for the same patient, only one referral per code is kept, and duplicates are removed. This study included all OB/GYN referral requests submitted through the e-referral system between January 2023 and December 2024 with complete data documentation, while reports with incomplete data documentation were excluded. Data were screened for erroneous or implausible values, and none were identified. Referrals with incomplete documentation (e.g., bed type, requested subspecialty, and others) were excluded. Approximately 3% of records (*n* = 1222) had incomplete documentation and were excluded, yielding a final analytic sample of 39,526 referrals.

### 2.3. Variables and Measurements

Three categories of variables were examined in this study: demographic, geographic, and referral-specific characteristics. Geographic analysis focused on referral patterns across the five BUs. Referral-specific variables included: (1) OB/GYN subspecialty classification (general obstetrics and gynecology, obstetrics and fetal medicine, reproductive endocrinology and infertility, female reproductive system tumors, women’s endoscopic diseases, and pelvic floor reconstruction and urological surgery); (2) referral type (routine outpatient, routine inpatient, urgent, lifesaving, and services). Urgent—for cases necessitating a referral within 72 h based on the treating physician’s clinical assessment of the patient’s condition, with adherence to the MRC’s protocol, lifesaving—for critical cases requiring an immediate referral based on the treating physician’s clinical assessment of the patient’s condition following the MRC’s protocol, and services—for patients requiring a temporary referral to conduct specialized investigations (e.g., scans) before returning to the referring facility; (3) bed type (e.g., outpatient, ward, and ICU); (4) referral outcome (acceptance or rejection); (5) referral direction (internal— the referring and receiving facilities are located within the same administrative region, or external—the referring and receiving facilities are located in different administrative regions); and (6) temporal indicators (month and year). For temporal analysis of referral direction over 24 months, we coded referral direction as a binary outcome variable (internal = 1, external = 0) and created a continuous temporal index variable representing study month (ranging from 1 for January 2023 to 24 for December 2024).

### 2.4. Statistical Analysis

Descriptive statistics included frequencies and percentages for categorical variables, with means and standard deviations for continuous variables. Adjusted referral rate per 10,000 was calculated using the Saudi census statistics from the Saudi General Authority for Statistics. We analyzed temporal trends, regional variations, and subspecialty-specific patterns. Bivariate relationships were assessed using chi-square tests for categorical variables and one-way ANOVA for continuous variables. Post hoc pairwise comparisons were performed using Bonferroni correction for multiple comparisons. Additionally, multivariable logistic regression examined temporal trends in referral direction over the 24-month study period, adjusting for potential confounders. Statistical significance was set at *p* < 0.05. Analyses were performed using Stata Statistical Software version 17 (StataCorp, College Station, TX, USA, 2021). Effect sizes were calculated using eta-squared (η^2^) for ANOVA and Cramér’s V for chi-square tests to assess the practical significance of observed associations (Supplementary Table S1).

### 2.5. Ethical Considerations

This study received ethical approval from the Institutional Review Board of the Saudi Ministry of Health (protocol code 23-77-E, approved 20 September 2023). The dataset was fully de-identified prior to analysis, with no personal identifiers that could link data to individual patients. The requirement for individual informed consent was waived due to the retrospective nature of the analysis and the use of de-identified data. All procedures adhered to the principles of the Declaration of Helsinki.

## 3. Results

This study analyzed 39,526 OB/GYN referral requests processed through the digital health surveillance and coordination platform during the period of 2023–2024, showing that middle-aged females, with a mean age of 36.47 ± 12.88, were the predominant group (Table 1). The findings revealed variations in the requested subspecialties, with General Obstetrics and Gynecology comprising the majority (60.68%) of the referrals, followed by obstetrics and fetal medicine (16.37%), while Pelvic Floor Reconstruction and Urological Surgery was the least (1.72%). Geographically, referrals originated from all BUs, with most requests from the Western and Central BUs (28.78% and 25.59%, respectively). The digital health surveillance and coordination platform achieved 91.81% overall acceptance rates, and the majority were referred internally (80.16%). Temporal analysis indicates an increase in referral volumes from 2023 (47.06%) to 2024 (52.94%).

The demand for each OB/GYN subspecialty varied based on the patient’s age (Table 2). Older females showed a higher need for ‘Pelvic Floor Reconstruction and Urological Surgery’ and Female Reproductive System subspecialities, with mean ages of 49.98 and 49.15 years, respectively. Conversely, Reproductive Endocrinology and Infertility was more common among younger females, with a mean age of 34.36 years. The other subspecialties were predominantly required by middle-aged females, with mean ages from 35.59 to 40.21 years. Post hoc analysis with Bonferroni correction confirmed significant differences between most subspecialty pairs (*p* < 0.05).

Table 3 details the referral characteristics based on the requested OB/GYN subspecialties. The analysis revealed distinct bed requirement patterns, with outpatient care predominating overall (71.35%). However, Obstetrics and Fetal Medicine demonstrated a unique profile with the highest demand for specialized inpatient services. Most notably, this subspecialty accounted for 55.62% of referrals requiring neonatal intensive care unit (NICU) beds. In contrast, other subspecialties showed minimal NICU requirements, with General OB/GYN accounting for only 3.78% and the remaining subspecialties showing negligible or no NICU utilization. Most referrals were for outpatient care (71.35%). Urgent referrals comprised 22.05% of the total, the highest for obstetrics and fetal medicine (58.85%). Referral acceptance rates were high overall (91.81%) but lowest for reproductive endocrinology and infertility (83.88%). Internal referrals dominated for most subspecialties, except for reproductive endocrinology and infertility, which had more external referrals (55.75%).

Table 4 presents the proportion and rate of subspecialty requests per 10,000 population across the five Saudi BUs, demonstrating significant regional specialization patterns (χ^2^ = 4865.30, *p* < 0.0001). Although the Western and Central BUs had the highest number of referral requests, they had the lowest referral rates in relation to population size (3.79 and 3.72 per 10,000, respectively). The Southern BU exhibited the highest rate of referral requests per 10,000 people (7.27), with notable dominance in General OB/GYN (48.87), Reproductive Endocrinology and Infertility (13.38), and Female Reproductive Tumors (3.55) subspecialties. The Western BU substantially recorded the highest rate in Obstetrics & Fetal Medicine (13.33), whereas the Central BU recorded the lowest (2.82). The Northern BU showed the highest rate for Women’s Endoscopic Diseases (1.61), while the Eastern BU demonstrated a predominance in Pelvic Floor Reconstruction (1.37).

**Figure 2:**
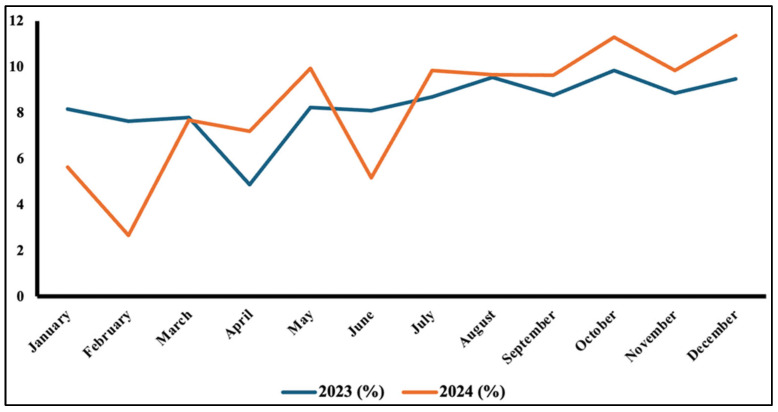
Temporal pattern of Obstetrics and Gynecology referrals in Saudi Arabia (2023–2024). Monthly distribution differed significantly between years (Pearson χ^2^ = 909.76, df = 11, *p* < 0.001).

The temporal analysis of the referrals revealed significant variation in utilization patterns throughout the study period (Figure 2, *p* < 0.0001). Monthly referral distribution differed significantly between 2023 and 2024 (Pearson χ^2^ = 909.76, df = 11, *p* < 0.001). The year 2023 showed a steady utilization of the e-referral service (from 7.63% to 8.85%), except in April, when the service utilization decreased to its lowest point (4.87%). In the following year, the e-referral requests exhibited significant fluctuations during the first two quarters, with a notable decline in February (2.68%), followed by an increase in volume during the fourth quarter, reaching a peak in December (11.38%).

Table 5 shows that after adjusting for potential confounders, the odds of internal referral significantly increased over time (OR = 1.017 per month; 95% CI: 1.013–1.020; *p* < 0.001), indicating a 1.7% increase in the likelihood of internal referral with each passing month over the 24-month study period.

## 4. Discussion

This study examined OB/GYN referral characteristics and trends across all subspecialties and BUs in Saudi Arabia from 2023 to 2024, specifically analyzing referral volumes, subspecialty distribution, geographic patterns, acceptance rates, and temporal variations through the MRC digital health surveillance and coordination platform. Our analysis of 39,526 national e-referral records is among the largest datasets for analyzing women’s healthcare access in the Middle East region. The findings revealed distinct subspecialty-specific utilization patterns that may inform healthcare capacity planning and resource allocation. The observed overall acceptance rate was 91.81%, with subspecialty variations in care coordination needs, with general OB/GYN dominating referral volumes (60.68%), Obstetrics and Fetal Medicine showing high urgent referral rates (58.85%) and the highest demand for specialized inpatient services including NICU utilization (55.62%), and Reproductive Endocrinology and Infertility exhibiting unique patterns with higher external referral rates (55.75%) and lower acceptance rates (83.88%). The observed proportion of internal referrals was 80.16%, with an overall acceptance rate of 91.81% in this dataset. Overall, these findings provide a descriptive overview of OB/GYN referral patterns that may inform capacity planning across Saudi Arabia, while also offering an analytical framework that could be adapted by other integrated healthcare systems seeking to evaluate their specialized women’s health service delivery. This study describes system-level referral patterns rather than ranking the performance of individual regions or facilities.

The digital surveillance platform captured a mean patient age of 36.47 ± 12.88 years, representing a notably higher demographic profile compared to most published studies on OB/GYN service utilization, which typically report mean ages in the mid-to-late twenties [36,37,38,39,40]. This higher mean age in our national referral surveillance data likely reflects several factors: the comprehensive nature of our digital platform dataset, including all OB/GYN subspecialties rather than focusing solely on obstetric or emergency care, and the inclusion of gynecological conditions that typically affect older reproductive-age women. The higher mean age also indicates potentially improved healthcare access, enabling women to seek specialized care later in their reproductive years [41,42]. These age distribution patterns captured through the surveillance system have implications for healthcare capacity planning, indicating opportunities to tailor subspecialty services for middle-aged women’s reproductive health needs, including gynecologic oncology and reproductive endocrinology services. Furthermore, this demographic shift toward older patients seeking specialized care highlights the importance of developing age-appropriate clinical protocols and resource allocation strategies within the digital coordination platform [43,44].

The digital surveillance platform revealed General OB/GYN’s dominance at 60.68%, nearly two times larger than Obstetrics and Fetal Medicine (16.37%) and Reproductive Endocrinology and Infertility (14.94%) combined. This distribution underscores the general OB/GYN’s critical gatekeeper function, managing a broad reproductive health spectrum from routine gynecological care to initial pregnancy management before subspecialty referral when complications arise [45]. The high proportion of Maternal-Fetal Medicine referrals reflects significant high-risk pregnancy management patterns, while the notable Reproductive Endocrinology volume suggests growing utilization of fertility services and complex hormonal disorders care. Compared to international patterns, this distribution aligns with healthcare systems emphasizing comprehensive primary gynecologic care with established subspecialty referral pathways [45,46]. These findings highlight the importance of balanced investment strategies: maintaining General OB/GYN workforce capacity to handle the majority caseload while ensuring subspecialty services can accommodate complex referrals. The digital platform may offer potential for refining decision-support tools for primary care providers and maintaining clear subspecialty referral criteria across service levels.

The predominance of outpatient department referrals (71.35%) is consistent with global trends toward ambulatory care optimization [47,48]. A recent McKinsey survey found a significant portion of healthcare visits could be effectively managed in outpatient settings, aligning with evidence showing appropriate outpatient care can reduce expenditures by 30–40% [49] while improving patient satisfaction [50]. The proportion of urgent referrals (22.05%) reflects the MRC system’s role in coordinating complex cases requiring advanced subspecialty services. These cases involve coordinated patient transfer to appropriate facilities, whether within the same administrative region or across regions, with Ministry of Health cost coverage. This referral pattern underscores the importance of clear protocols that prioritize complex cases requiring access to advanced medical centers and specialist expertise [51]. The data suggests that investment in telemedicine capabilities and remote consultation systems could further optimize urgent care coordination while reducing unnecessary patient transfers through enhanced pre-referral assessment [45,46,52].

The high utilization of NICU beds for Obstetrics & Fetal Medicine referrals (55.62%) underscores the complex nature of high-risk pregnancies and the critical importance of specialized NICU capacity. This finding aligns with international data showing that 10–15% of pregnancies require specialized obstetric care, with a significant proportion necessitating NICU services [53]. The corresponding high acceptance rate (96.65%) for these referrals suggests prioritization of resources for critical Maternal–Fetal Medicine cases, aligning with the objectives of the new model of care that prioritizes birth outcomes [54]. Conversely, the near-exclusive outpatient utilization for Reproductive Endocrinology and Infertility (99.26%) reflects the ambulatory nature of fertility treatments and evaluations, mirroring patterns observed in developed healthcare systems worldwide [41,42].

Internal referral odds increased 1.7% monthly, suggesting progressive strengthening of regional coordination capacity. Furthermore, the predominance of internal referrals across most subspecialties suggests that most specialized care needs in this dataset were met within regional networks, a pattern consistent with integrated healthcare delivery models [54,55]. This internal referral pattern demonstrates that most specialized care needs can be met within regional networks, reducing patient burden and healthcare costs. The higher external referral rate for Reproductive Endocrinology and Infertility (55.75%) reflects the current strategic distribution of these highly specialized fertility services, where centers of excellence enable concentration of expertise and advanced reproductive technologies while maintaining quality and accessibility through the coordinated MRC referral system. This pattern is consistent with international models where sophisticated equipment and specialized personnel are concentrated in designated centers [56]. As the healthcare system continues to evolve, the external referral pattern for Reproductive Endocrinology and Infertility services represents one model for delivering complex care, while the predominantly internal referral patterns observed in other subspecialties suggest regional capacity for service provision. However, centralization of specialized services, while potentially improving outcomes through higher case volumes, may introduce access considerations related to patient travel burden and waiting times that were not measured in this study and warrant further investigation using formal equity metrics such as geographic distance, socioeconomic status, and time-to-acceptance.

The overall high acceptance rate (91.81%) in this dataset is comparable with international benchmarks for specialist care access [57,58]. This observed acceptance proportion aligns with Saudi Arabia’s healthcare transformation initiatives [55]. The subspecialty-specific variations reveal important patterns, with reproductive endocrinology and infertility’s acceptance rate (83.88%) likely reflecting clinical screening criteria. The higher non-acceptance proportion may result from patients not meeting eligibility criteria, including age restrictions, previous pregnancy history, duration of infertility, or completion of preliminary investigations; however, the clinical appropriateness of individual referral decisions cannot be determined from administrative data alone [59,60], with some cases possibly serviceable locally. Age-related prognosis considerations, with in vitro fertilization (IVF) success rates showing substantial decline after age 40 and particularly poor outcomes after age 43, may inform eligibility assessments [61,62]. Enhanced pre-referral checklists and communication protocols could improve the referral process by reducing avoidable non-acceptance when clinical parameters are not properly communicated at the referring facility level.

This analysis of the MRC digital referral platform describes how an integrated e-referral system coordinates specialized women’s health services across a large national healthcare network. The observed referral patterns, including high acceptance rates across subspecialties and coordination of both routine ambulatory care and time-sensitive urgent referrals through standardized protocols, illustrate the operational scope of the platform. These findings contribute to the growing understanding of technology-enabled referral systems within modern healthcare delivery. The MRC framework may offer practical insights for other healthcare systems considering similar digital coordination approaches.

### Study Strengths and Limitations

This study demonstrates several methodological strengths. The analysis of nearly 40,000 referral records across a 24-month period represents one of the largest datasets examining OB/GYN e-referral coordination nationally, providing a robust empirical foundation. The inclusion of multiple OB/GYN subspecialties offers a broad view of women’s healthcare delivery across the Kingdom of Saudi Arabia. The study benefits from systematic data collection through the established MRC digital infrastructure, ensuring consistent capture across regions and reducing selection bias. Additionally, the integration of acceptance rates, referral volumes, bed type requirements, and geographic distribution provides multidimensional insights into referral patterns.

However, several limitations should be acknowledged. First, the study design limits the ability to establish causal relationships between observed patterns and underlying factors influencing referral decisions. Second, as a secondary analysis of administrative data, the study is inherently constrained by the variables captured within the MRC platform, which do not encompass the full range of factors that may influence referral patterns and access. Third, the analysis operates at the system level and does not incorporate multilevel modeling that simultaneously examines patient-level and facility-level determinants. Fourth, this study focused on the referral coordination process—specifically, how cases are submitted, classified, and accepted—rather than tracking post-referral clinical trajectories; accordingly, measures such as time-to-appointment, clinical outcomes, and patient satisfaction were beyond the current scope. Finally, the focus on referral patterns alone does not provide insights into primary care management or patient pathways prior to specialist referral, which could inform a more complete understanding of the healthcare continuum.

Despite these limitations, this study establishes an essential baseline characterization of national OB/GYN e-referral coordination that can guide future investigations. Future studies should employ advanced time-series analyses to examine temporal trends in e-referral utilization, link referral data with electronic health records to assess downstream clinical outcomes, including time-to-appointment and post-referral health trajectories, and investigate patient and provider factors influencing OB/GYN referral rejection to inform system performance improvement. Additionally, future research should investigate regional OB/GYN services through primary data collection, employing multilevel study designs that incorporate patient-level socioeconomic variables, facility-level characteristics, and regional-level factors for a more comprehensive understanding of referral patterns and outcomes.

## 5. Conclusions

This study provides a comprehensive description of OB/GYN referral patterns through the MRC digital referral platform across Saudi Arabia’s healthcare system. The observed acceptance rate was high (91.81% overall) across diverse subspecialty needs, from routine reproductive endocrinology consultations to urgent maternal-fetal medicine cases requiring immediate NICU access. The majority of referrals were for outpatient care (71.35%), with urgent referrals constituting 22.05% of cases. Subspecialty-specific utilization patterns were observed, with obstetrics and fetal medicine demonstrating the highest acceptance rates (96.65%) and reproductive endocrinology services showing lower acceptance (83.88%), likely reflecting eligibility criteria. Internal referral odds increased 1.7% monthly over the study period, indicating progressive strengthening of regional coordination capacity. The predominance of internal referrals across most subspecialties suggests regional service capacity, while external referrals for specialized fertility services reflect the concentration of these services. These descriptive findings provide a system-level overview that may inform capacity planning; however, future studies incorporating clinical outcomes, patient-reported measures, and time-to-decision metrics are needed to fully evaluate the quality and accessibility of OB/GYN care coordination.

## Figures and Tables

**Table 1:** Demographic and Distribution Characteristics of Obstetrics and Gynecology Referrals in Saudi Arabia (2023–2024).

Characteristics	Mean	SD
Age	36.86	10.65
Specialty/Subspecialty	Frequency (N)	Percentage (%)
General Obstetrics and Gynecology	23,983	60.68
Obstetrics and Fetal Medicine	6472	16.37
Reproductive Endocrinology and Infertility	5907	14.94
Female Reproductive System Tumors	1722	4.36
Women’s Endoscopic Diseases	764	1.93
Pelvic Floor Reconstruction and Urological Surgery	678	1.72
Business Unit (BU)		
Western BU	11,377	28.78
Central BU	10,115	25.59
Southern BU	8320	21.05
Eastern BU	5489	13.89
Northern BU	4225	10.69
Referral Outcome		
Accept	36,288	91.81
Reject	3238	8.19
Referral Direction		
Internal	31,685	80.16
External	7841	19.84
Year		
2023	18,602	47.06
2024	20,924	52.94
Total	39,526	100.00

Note: N = frequency; % = percentage. Data collected from the Saudi Medical Referral Center (MRC) system from 2023 to 2024.

**Table 2:** Age Distribution Across Obstetrics and Gynecology Subspecialties Referrals in Saudi Arabia (2023–2024).

Subspecialty	Mean (Years)	SD (Years)	*p*-Value
General Obstetrics and Gynecology	35.59	13.21	<0.001
Obstetrics and Fetal Medicine	36.47	12.88	
Reproductive Endocrinology and Infertility	34.36	6.12	
Female Reproductive System Tumors	49.15	15.56	
Women’s Endoscopic Diseases	40.21	11.25	
Pelvic Floor Reconstruction and Urological Surgery	49.98	12.90	

Note: SD = Standard Deviation. Significant age differences across subspecialties (ANOVA: *p* < 0.001). Bonferroni post hoc analysis: all pairwise comparisons were significant (*p* < 0.001) except Female Reproductive System Tumors vs. Pelvic Floor Reconstruction and Urological Surgery (*p* = 1.000) and Reproductive Endocrinology and Infertility vs. Obstetrics and Fetal Medicine (*p* = 0.189).

**Table 3:** Referral Characteristics by Obstetrics and Gynecology Subspecialty in Saudi Arabia (2023–2024).

Characteristics	General OB/GYN N (%)	Obstetrics and Fetal Medicine N (%)	Reproductive Endocrinology and Infertility N (%)	Female Reproductive Tumors N (%)	Women’s Endoscopic Diseases N (%)	Pelvic Floor Reconstruction N (%)	Total N (%)	*p*-Value (χ^2^, df)
Bed Type								<0.0001 * (χ^2^ = 17,372, df = 30)
OPD	17,156 (71.53%)	2431 (37.56%)	5863 (99.26%)	1418 (82.35%)	707 (92.54%)	639 (94.25%)	28,203 (71.35%)	
Ward Bed	5572 (23.23%)	421 (6.50%)	42 (0.71%)	303 (17.60%)	57 (7.46%)	39 (5.75%)	6434 (16.28%)	
NICU	906 (3.78%)	3600 (55.62%)	0 (0.00%)	0 (0.00%)	0 (0.00%)	0 (0.00%)	4506 (11.40%)	
ICU	291 (1.21%)	19 (0.29%)	1 (0.02%)	0 (0.00%)	0 (0.00%)	0 (0.00%)	311 (0.79%)	
PICU	43 (0.18%)	0 (0.00%)	1 (0.02%)	0 (0.00%)	0 (0.00%)	0 (0.00%)	44 (0.11%)	
CCU	15 (0.06%)	1 (0.02%)	0 (0.00%)	1 (0.06%)	0 (0.00%)	0 (0.00%)	17 (0.04%)	
Referral Type								<0.0001 * (χ^2^ = 8200, df = 20)
Routine OPD	17,036 (71.03)	2391 (36.94)	5861 (99.22)	1347 (78.22)	704 (92.15)	634 (93.51)	27,973 (70.77)	
Urgent	4611 (19.23)	3809 (58.85)	5 (0.08)	234 (13.59)	32 (4.19)	23 (3.39)	8714 (22.05)	
Routine Inpatient	1540 (6.42)	221 (3.41)	36 (0.61)	116 (6.74)	25 (3.27)	11 (1.62)	1949 (4.93)	
Life Saving	604 (2.52)	37 (0.57)	0 (0.00)	15 (0.87)	1 (0.13)	3 (0.44)	660 (1.67)	
Services	192 (0.80)	14 (0.22)	5 (0.08)	10 (0.58)	2 (0.26)	7 (1.03)	230 (0.58)	
Referral Outcome								<0.0001 * (χ^2^ = 762, df = 5)
Accept	22,208 (92.60)	6255 (96.65)	4955 (83.88)	1544 (89.66)	671 (87.83)	655 (96.61)	36,288 (91.81)	
Reject	1775 (7.40)	217 (3.35)	952 (16.12)	178 (10.34)	93 (12.17)	23 (3.39)	3238 (8.19)	
Referral Direction								<0.0001 * (χ^2^ = 9400, df = 5)
Internal	22,713 (94.70)	4070 (62.89)	2614 (44.25)	1133 (65.80)	576 (75.39)	579 (85.40)	31,685 (80.16)	
External	1270 (5.30)	2402 (37.11)	3293 (55.75)	589 (34.20)	188 (24.61)	99 (14.60)	7841 (19.84)	
Total	23,983 (60.68)	6472 (16.37)	5907 (14.94)	1722 (4.36)	764 (1.93)	678 (1.72)	39,526 (100.00)	

N (%) = frequency (column percentage); OB/GYN = Obstetrics and Gynecology; OPD = Outpatient Department; NICU = Neonatal Intensive Care Unit. Within the MRC system, NICU bed referrals predominantly involve high-risk pregnancies with anticipated preterm delivery (<30 weeks of gestation). ICU = Intensive Care Unit; PICU = Pediatric Intensive Care Unit for patients < 14 years old. Data collected from the Saudi Medical Referral Center (MRC) system from 2023 to 2024. * = Denotes statistical significance.

**Table 4:** Distribution of Obstetrics and Gynecology Subspecialties by Business Unit in Saudi Arabia (2023–2024).

BU	General OB/GYN		Obstetrics & Fetal Medicine		Reproductive Endocrinology and Infertility		Female Reproductive Tumors		Women’s Endoscopic Diseases		Pelvic Floor Reconstruction		Total		*p*-Value
	N (%)	Rate per 10,000	N (%)	Rate per 10,000	N (%)	Rate per 10,000	N (%)	Rate per 10,000	N (%)	Rate per 10,000	N (%)	Rate per 10,000	N (%)	Rate per 10,000	
Central BU	6941 (28.94)	25.53	767 (11.85)	2.82	1492 (25.26)	5.49	342 (19.86)	1.26	260 (34.03)	0.96	313 (46.17)	1.15	10,115 (25.59)	3.72	<0.0001 *
Eastern BU	3115 (12.99)	24.06	665 (10.28)	5.14	1082 (18.32)	8.36	316 (18.35)	2.44	133 (17.41)	1.03	178 (26.25)	1.37	5489 (13.89)	4.24	
Western BU	5743 (23.95)	19.13	4001 (61.82)	13.33	1029 (17.42)	3.43	468 (27.18)	1.56	91 (11.91)	0.30	45 (6.64)	0.15	11,377 (28.78)	3.79	
Northern BU	2588 (10.79)	36.58	494 (7.63)	6.98	772 (13.07)	10.91	190 (11.03)	2.69	114 (14.92)	1.61	67 (9.88)	0.95	4225 (10.69)	5.97	
Southern BU	5596 (23.33)	48.87	545 (8.42)	4.76	1532 (25.94)	13.38	406 (23.58)	3.55	166 (21.73)	1.45	75 (11.06)	0.65	8320 (21.05)	7.27	
Total	23,983 (60.68)	27.04	6472 (16.37)	7.30	5907 (14.94)	6.66	1722 (4.36)	1.94	764 (1.93)	0.86	678 (1.72)	0.76	39,526 (100.00)	4.46	

Note: N (%) = frequency (column percentage); OB/GYN = Obstetrics and Gynecology; BU = Business Unit. Data collected from the Saudi Medical Referral Center (MRC) system from 2023 to 2024. Rate per 10,000 was calculated using the latest statistics from the Saudi General Authority for Statistics, released for the year 2022 [35]. Chi-square test of independence comparing subspecialty distribution across business units (χ^2^ = 4900, df = 20). * = Denotes statistical significance.

**Table 5:** Multivariable Logistic Regression Analysis of Factors Associated with Internal Referral Direction (N = 39,528).

Variables	Odds Ratio	95% CI	*p*-Value
Temporal Trend			
Study month (per month increase)	1.017	1.013–1.020	<0.001
Demographics			
Age (per year increase)	1.025	1.023–1.027	<0.001
Gender (female vs. male)	0.803	0.598–1.078	0.144
Administrative Region			
Central BU	Reference	–	–
Eastern BU	0.04	0.034–0.048	<0.001
Western BU	0.056	0.047–0.067	<0.001
Northern BU	0.017	0.015–0.021	<0.001
Southern BU	0.041	0.034–0.049	<0.001
Referral Type			
Routine Inpatient	Reference	–	–
Routine Outpatient	0.435	0.378–0.502	<0.001
Life/Organ Saving	3.604	2.394–5.426	<0.001
Emergency	0.708	0.608–0.824	<0.001
Services	1.727	1.063–2.807	0.027

Abbreviations: CI, confidence interval; BU, Business Unit. Note: Internal referral coded as 1, external referral coded as 0. Model adjusted for all variables shown.

## Data Availability

The data used in this study contains sensitive health information and is subject to national data protection laws, thus restricting public sharing. Aggregated or anonymized data may be made available upon reasonable request.

## Supplementary Materials

The following supporting information can be downloaded at: https://www.mdpi.com/article/10.3390/healthcare14070883/s1, Supplementary Table S1. Effect size analysis for OB/GYN referral associations (N = 39,526).

## Abbreviations

The following abbreviations are used in this manuscript:

BU	Business Unit
CCU	Coronary Care Unit
ICU	Intensive Care Unit
MRC	Medical Referral Center
N	Number/Frequency
NICU	Neonatal Intensive Care Unit
OB/GYN	Obstetrics and Gynecology
OPD	Outpatient Department
PICU	Pediatric Intensive Care Unit
SD	Standard Deviation
%	Percentage
