# Supplementary material for: Healthcare Access Through Digital Coordination: A Nationwide Analysis of Obstetrics and Gynecology E-Referral Patterns in Saudi Arabia
Source: Healthcare (Basel). 2026 Mar 30;14(7):883. doi: 10.3390/healthcare14070883 (PMC13073936; doi:10.3390/healthcare14070883)
Supplement: Supplementary file 1 [file healthcare-14-00883-s001.zip › healthcare-4171488-supplementary.pdf]

Supplementary table S1. Effect size analysis for OB/GYN referral associations (N = 39,526)

Source	Analysis	Test Statistic	df	p-value	Effect Size	Interpretation
**Table 2**						
	Age × Subspecialty	F = 941.93	5	< 0.001	$\eta^2 = 0.30$	Large
**Table 3**						
	Bed Type × Subspecialty	$\chi^2 = 17{,}372$	30	< 0.001	V = 0.30	Moderate
	Referral Type × Subspecialty	$\chi^2 = 8{,}200$	20	< 0.001	V = 0.23	Moderate
	Referral Outcome × Subspecialty	$\chi^2 = 762$	5	< 0.001	V = 0.14	Small
	Referral Direction × Subspecialty	$\chi^2 = 9{,}400$	5	< 0.001	V = 0.49	Large
	Region × Subspecialty	$\chi^2 = 4{,}900$	20	< 0.001	V = 0.18	Small
**Figure 2**						
	Months × Year	$\chi^2 = 910$	11	< 0.001	V = 0.15	Small
Note: Effect size interpretation — η^2 (Eta-squared) for ANOVA: ≥ 0.14 large, 0.06–0.13 medium, < 0.06 small; Cramér's V for Chi-square: > 0.40 large, 0.20–0.40 moderate, 0.10–0.20 small, < 0.10 negligible						
